# Side-of-Implantation Effect on Functional Asymmetry in the Auditory Cortex of Single-Sided Deaf Cochlear-Implant Users

**DOI:** 10.1007/s10548-022-00902-3

**Published:** 2022-06-07

**Authors:** Anna Weglage, Verena Müller, Natalie Layer, Khaled H. A. Abdel-Latif, Ruth Lang-Roth, Martin Walger, Pascale Sandmann

**Affiliations:** 1 Faculty of Medicine and University Hospital Cologne, Department of Otorhinolaryngology, Head and Neck Surgery, Audiology and Pediatric Audiology, Cochlear Implant Center, University of Cologne, Cologne, Germany; 2 Jean-Uhrmacher-Institute for Clinical ENT Research, University of Cologne, Cologne, Germany

**Keywords:** Single-sided deafness, Cochlear implants, Event-related potential, Oddball paradigm, Hemispheric asymmetry, Cortical plasticity

## Abstract

Cochlear implants (CIs) allow to restore the hearing function in profoundly deaf individuals. Due to the degradation of the stimulus by CI signal processing, implanted individuals with single-sided deafness (SSD) have the specific challenge that the input highly differs between their ears. The present study compared normal-hearing (NH) listeners (N = 10) and left- and right-ear implanted SSD CI users (N = 10 left, N = 9 right), to evaluate cortical speech processing between CI- and NH-ears and to explore for side-of-implantation effects. The participants performed a two-deviant oddball task, separately with the left and the right ear. Auditory event-related potentials (ERPs) in response to syllables were compared between proficient and non-proficient CI users, as well as between CI and NH ears. The effect of the side of implantation was analysed on the sensor and the source level. CI proficiency could be distinguished based on the ERP amplitudes of the N1 and the P3b. Moreover, syllable processing via the CI ear, when compared to the NH ear, resulted in attenuated and delayed ERPs. In addition, the left-ear implanted SSD CI users revealed an enhanced functional asymmetry in the auditory cortex than right-ear implanted SSD CI users, regardless of whether the syllables were perceived via the CI or the NH ear. Our findings reveal that speech-discrimination proficiency in SSD CI users can be assessed by N1 and P3b ERPs. The results contribute to a better understanding of the rehabilitation success in SSD CI users by showing that cortical speech processing in SSD CI users is affected by CI-related stimulus degradation and experience-related functional changes in the auditory cortex.

**Supplementary Information:**

The online version contains supplementary material available at 10.1007/s10548-022-00902-3.

## Introduction

Cochlear implants (CIs) can (partially) restore the hearing of individuals with severe to profound sensorineural hearing loss by direct electrical stimulation of the auditory nerve (Zeng et al. 2011). Compared to normal acoustic hearing, the sounds transmitted by a CI are limited in the spectral and temporal domain and have a smaller dynamic range (Drennan, 2008). Therefore, CI recipients need to adapt to the highly artificial inputs after implantation. However, the speech understanding with a CI remains limited and highly variable across the patients (Lenarz et al. 2012; Roberts et al. 2013). Previous studies have shown that different individual factors contribute to this variability in CI outcome, among them peripheral factors (e.g. positioning of the electrode array), cortical reorganisation as induced by auditory deprivation (before cochlear implantation) and by the (limited) electrical hearing with a CI (after cochlear implantation) (Lazard et al. 2012b; Lazard et al. 2012a, b, c).

The clinical margins for CI indication have been extended over the last years, now including single-sided deaf (SSD) individuals (Arndt et al. 2011a, b; Buechner et al. 2010). SSD CI users are to be distinguished from bilateral (CI on both ears) and bimodal (CI on one ear and hearing aid on the contralateral ear) CI users, since in SSD CI users the signal quality of the input highly differs between the two ears, and the normal-hearing (NH) ear typically remains the dominant communication channel. This leads to maximal asymmetric auditory processing in this group of patients (Gordon et al. 2013; Kral et al., 2013). Nevertheless, Arndt et al. (2011a, b) showed improved hearing abilities in SSD patients aided with a CI compared to alternative treatments like contralateral routing of signal (CROS) or bone-anchored hearing aids (BAHA). SSD CI patients particularly benefit in sound localisation, speech understanding in noise and quality of life due to the restored binaural hearing (Kitterick et al. 2015). However, it remains not well understood whether the extent of the benefits in SSD CI users depends on the side of implantation. Similar to the findings from CI users with bilateral hearing loss (Mosnier et al. 2014), first results point to a right-ear advantage for speech recognition ability in SSD CI patients as well (Wettstein and Probst, 2018). In addition to the largely unresolved question regarding the side-of-implantation effects, it remains unclear whether SSD patients—when tested with only the NH ear—show the same speech processing capabilities as NH listeners who use only one of their ears (Arndt et al. 2019; Maslin et al. 2015). To better understand these effects on the CI outcome in SSD CI users, the current study systematically compared speech processing between left- and right-ear implanted SSD users on the one hand, and between these two patient groups and NH listeners on the other hand.

After cochlear implantation, speech intelligibility is typically assessed via behavioural measures, in particular word and sentence tests (Haumann et al. 2010; Hey et al. 2016, 2014; Hahlbrock, 1953; Hochmair-Desoyer et al. 1997). Event-related potentials (ERPs) however allow the *objective* evaluation of speech processing in CI users with a high temporal resolution (Luck, 2014). Previous studies have used ERPs in the electroencephalogram (EEG) to study cortical auditory processing in CI users (Beynon et al. 2005; Finke et al. 2016; Finke et al. 2015; Henkin et al. 2009; Sandmann et al. 2010; Sandmann et al. 2009; Groenen et al. 2001). Most of these studies applied an auditory oddball paradigm, in which a frequent standard sound and an infrequent deviant sound were pseudo-randomly presented, meaning that a deviant sound is followed by at least two standard sounds. Using this type of paradigm allows the study of the N1 ERP (negativity around 100 ms post stimulus) and the P2 ERP (positivity around 200–250 ms post stimulus). These ERPs are elicited in response to both the standard and deviant sounds and originate mainly from the auditory cortex (Crowley and Colrain, 2004; Näätänen and Picton, 1987). An additional deviant-related P3b response (positivity around 300–650 ms) is elicited if the central auditory system can discriminate between the standard and the deviant sound (Henkin et al. 2009). It has been widely assumed that the P3b reflects the evaluation and classification of incoming auditory events (for a review, see Polich, 2007).

Most of the previous EEG studies on CI users have used an auditory oddball paradigm to study N1, P2 and P3b ERPs in individuals with *bilateral* hearing loss (Beynon et al. 2005; Finke et al. 2016, 2015; Henkin et al. 2009). ERPs of postlingually deafened adult CI users seem to be reduced in amplitude and prolonged in latency when compared to NH listeners, suggesting that CI users have difficulties in the sensory (N1, P2) and higher-level cognitive processing (P3b) of the limited CI input (Beynon et al. 2005; Finke et al. 2016; Henkin et al. 2014; Henkin et al. 2009; Sandmann et al. 2009). Moreover, adult CI users with bilateral hearing impairment show functional changes in the auditory cortex contra- and ipsilateral to the CI ear after implantation (Finke et al. 2015; Green et al. 2005). It is therefore not surprising that this group of patients can show altered functional asymmetry in the auditory cortex when compared to NH listeners, suggesting that auditory deprivation and/or cochlear implantation induce changes in the normal pattern of cortical response asymmetries. In contrast to implanted children with SSD (Lee et al. 2020; Polonenko et al. 2017), not much is known about functional changes in the adult auditory cortex of SSD CI patients. Knowledge about plasticity in the ipsi- and contralateral auditory cortex in SSD patients could help to understand the factors contributing to the CI outcome in these individuals. Thus, one principal aim of the present study was to evaluate the side-of-implantation effect on the functional asymmetry in the auditory cortex of adult SSD CI users.

Most of the previous studies using the oddball paradigm have been restricted to one stimulus pair (Billings et al. 2011; Sasaki et al. 2009). However, it is of clinical interest to develop a time-optimized multi-deviant oddball paradigm, which allows assessing multi-attribute auditory discrimination ‘profiles’. In the present study, we used a two-deviant oddball paradigm with one standard syllable and two types of deviant syllables of different acoustic–phonetic demand. In addition to NH controls, left- and right-ear implanted SSD CI users were tested sequentially on both ears. We systematically compared the behavioural and electrophysiological measures within and between the different groups of adult participants, which allowed us to address the following research questions:

1. Can a two-deviant oddball paradigm be used to objectively evaluate the speech discrimination ability in SSD CI users?
2. Do SSD CI users show differences in speech processing between the CI ear and the NH ear?
3. Is there a side-of-implantation effect on speech processing via the *CI ear* in SSD CI users?
4. Is there a side-of-stimulation effect on speech processing via the *NH ear* in SSD CI users and in NH listeners?

Following recent results on adult SSD CI users, we expected differences in behavioural and ERP measures between the CI ear and the NH ear in SSD CI users (Bönitz et al. 2018; Finke et al. 2016). In accordance with previous observations on the CI users with *bilateral* hearing loss, we hypothesised an altered functional asymmetry in the auditory cortex of SSD CI users when compared to NH listeners (Sandmann et al. 2009).

## Material and Methods

### Participants

Nineteen single-sided post-lingually deafened CI users participated in this study (six male; two left handers). All of them had no history of neurological or psychiatric disorders. All participants used their CI at a daily basis (15.5 ± 0.7 h/day) for at least ten months (mean = 18 months; sd = 8 months). The age ranged from 37 to 66 years (mean = 53.26 years; sd = 8.49 years). The duration of deafness before implantation varied from two months to 36 years (mean = 69 months; sd = 115 months). Since this variable is difficult to determine, anamnestic conversations were used to determine the time point at which a conventional hearing aid was no longer sufficient to understand speech. The duration of deafness was then calculated as the period between this time point and the cochlear implantation. All subjects were unilaterally implanted with a CI, nine of them on the right side and ten on the left side. Apart from two participants, all of the CI users were right-handed, as assessed by the Edinburgh inventory (Oldfield, 1971). According to previous studies with SSD CI users (Bönitz et al. 2018; Finke et al. 2016), the four pure tone average (4PTA–over 0.5, 1,2 and 4 kHz) of the contralateral NH ear was ≤ 30 dB. Speech comprehension was tested using the Freiburg monosyllabic word test (Hahlbrock, 1970) and the Oldenburg sentence test (Wagener et al. 1999), the latter conducted with and without background noise. Here, all stimuli were presented via a loudspeaker placed at a distance of 1.6 m from the listeners head located at 0° in a soundproofed booth. Additionally, ten age-matched NH controls participated in this study (two male). Their age ranged from 41 to 70 years (mean = 53.2 years; sd = 9.37 years). Detailed information about the implant systems and the demographic variables of the participants can be found in Table 1.

**Table 1:** Demographic variables, audiologic information and implant information of the participants

ID	Age [years]	Handed-ness	CI side	Group	Processor	Etiology (details)	Duration deafness [months]	CI use [months]	PTA CI ear [dB]	PTA NH ear [dB]	Freiburg Monosyllabic test (65 dB) [%]	OLSA quiet [%]	OLSA noise [dB]
vp03	51	Right	Right	Non-proficient	CP910	Morbus meniere	19	24	32.5	19.75	75	96	− 3.2
vp04	38	Right	Right	Proficient	Opus	Sudden deafness	12	36	31.5	3.5	40	97	1.4
vp06	62	Right	Right	Non-proficient	CP910	Cholesteatoma surgery	312	15	34.25	30	30	47	20.1
vp10	43	Right	Right	Non-proficient	CP910	Sudden deafness	12	19	25.75	14.5	65	89	−0.7
vp11	63	Right	Right	Proficient	CP910	Otitis media	18	12	34.25	14.75	10	23	Not applicable
vp14	61	Right	Right	Non-proficient	CP1000	Sudden deafness	7	11	24	15	65	90	− 0.4
vp15	55	Right	Right	Non-proficient	CP1000	Cogan 1 syndrome	3	10	22.5	10	95	99	− 2.6
vp18	49	Right	Right	Proficient	Sonnet	Otosclerosis	48	17	32.75	23.75	80	77	1.2
vp19	37	Right	Right	Non-proficient	Sonnet	Unknown	26	19	38.5	29	30	91	-0.7
vp01	58	Right	Left	Non-proficient	CP910	Sudden deafness	84	25	26	12	65	95	− 0.7
vp02	56	Left	Left	Proficient	CP910	Sudden deafness	30	12	36.5	4.25	10	7	Not applicable
vp05	48	Right	Left	Proficient	CP910	Sudden deafness	144	32	23.5	12	100	98	− 1.1
vp07	55	Right	Left	Non-proficient	CP910	Sudden deafness	432	20	43.5	8	55	64	4.3
vp08	53	Left	Left	Proficient	CP910	Sudden deafness	5	32	33.75	15.75	50	79	2.4
vp09	57	Right	Left	Non-proficient	CP910	Sudden deafness	21	10	31.25	11.5	55	92	− 1.2
vp12	66	Right	Left	Non-proficient	CP910	Unknown	120	15	29.5	30	70	94	0.5
vp13	63	Right	Left	Proficient	CP1000	Stapes surgery	19	10	37	12.5	55	75	0.7
vp16	54	Right	Left	Proficient	Sonnet	Acute hearing loss	10	14	28.75	16	80	100	− 2.6
vp17	43	Right	Left	Proficient	CP1000	Petrous bone fracture	2	11	22.5	6.25	55	78	6.1

### Stimuli

The stimuli consisted of three different syllables which were taken from the Oldenburg logatome (OLLO) corpus, providing natural spoken language stimuli (Welge-Lüßen et al. 1997). The stimuli were generated by cutting the syllables /ki/, /ti/ and /ka/ out of the available logatomes from one speaker (male speaker 2, V6 “normal spelling style” and N3 “dialect”). All syllables had a duration of 300 ms and were normalised using the RMS function of the Adobe Audition software. The syllables differed by the place of articulation in the consonant contrast (/k/ vs. /t/) (Henkin et al. 2009) and by phonetic features in the vowel contrast (/a/ vs. /i/), in particular the vowel height and the vowel place (Micco et al. 1995). The German vowels /i/ and /a/ differ in the central frequencies of the first (F1) and second formant (F2). The formant values of /a/ are 730 Hz for F1 and 1284 Hz for F2. Regarding the vowel /i/, the formant values are 278 Hz (F1) and 2139 Hz (F2). In the German language, these formant values indicate the highest contrast between vowels, which should be perceivable by most of the CI users (Groenen et al. 2001). Unlike the aforementioned vowels, the contrast of the consonants /k/ vs. /t/ is very small, only differing in their place of articulation. The syllables /ki/ and /ti/ differ in rapid spectral changes in the transition of F2, which represents the articulatory movement from the consonant to the vowel (Kent, 1997). Those characteristics are very difficult to distinguish for CI users*.* In this study, we deliberately used one easier (/ki/ vs. /ka/) and one more difficult stimulus contrast (/ki/ vs. /ti/) to study the effects of auditory discrimination ability on behavioural and ERP measures in SSD CI patients.

In addition to the auditory oddball task with “original”, unprocessed syllables, the NH control group performed three additional blocks with degraded, “vocoded” syllables. This adjusted sound condition allowed to analyse the effects of stimulus degradation comparable to CI processing (Shannon et al. 1995). A noise vocoder was used to degrade the syllables (Gaudrain and Başkent, 2015). The MATLAB code is available online (see Gaudrain, 2016). The vocoder filtered the signal into four bands using 12th order, zero-phase Butterworth bandpass filters. The band boundaries were equally spaced based on a 35-mm basilar membrane distance (Greenwood, 1990) across a frequency range between 0.2 and 20 kHz. To extract the temporal envelope, the output of each band was half-wave rectified and low-pass filtered at 250 Hz (zero-phase fourth order Butterworth filter). The envelope was then multiplied by a wide-band noise carrier, and the resulting signal was summed across bands.

### Task and Procedure

The experimental paradigm consisted of an auditory oddball task. The participants were presented with a frequent standard syllable (/ki/, 80% probability) and two infrequent deviant syllables (/ka/ and /ti/, 10% probability each). The participants were instructed to respond to deviant syllables via a button press of a computer mouse. The total of 800 trials were separated into three blocks with reasonable breaks in between. If a participant showed short response times, the inter-stimulus interval of 1400 ms was shortened accordingly. Hence, the measurement time added up to a maximum of 19 min (800 trials × 1400 ms) per ear. The stimuli were presented in a pseudo-randomised order with the constraint that a deviant syllable was preceded by at least three standard syllables. This was, however, not known by the participants. Prior to the experiment, a short training was performed at each ear. The participants sat in a comfortable chair in a sound attenuated booth. To avoid eye movements, the participants were instructed to look at a fixation cross on a computer monitor throughout the task.

In the *CI-only listening condition*, the processor of the CI users was put inside an aqua case from the manufacturer Advanced Bionics (https://www.advancedbionics.com) to avoid an additional stimulation of the NH ear. An earphone was inserted through a hole of the aqua case, where it was directly positioned over the microphone input of the CI. In general, all processors only fit into the aqua case with the compact batteries, which were provided by the clinic for each measurement. A long coil cable was used to connect the processor to the implant.

In SSD CI users, the use of an aqua case (in combination with an insert earphone) is advantageous compared to the stimulation via *direct connect* or loudspeakers for the following two reasons. First, the patients used CI processors from different manufacturers. A presentation of the stimuli via *direct connect* was not used to avoid an additional potential bias through the different ways of stimulus transfer into the different types of sound processors. Second, stimulus delivery via a *loudspeaker* would have been inappropriate, as this condition prevents a sufficient (passive) stimulus masking of the NH (i.e., non-tested) ear (Park et al. 2021).

Regarding the *NH-only condition* in SSD CI users and NH listeners, the stimuli were presented via inserted earphones positioned in the external auditory canal. The contralateral ear, that is, non-tested ear, was masked with an earplug in all conditions and groups. In general, the audio input level was calibrated to an acoustical input at 65 dB SPL. In addition, the participants performed a subjective rating before the start of the experiment. The loudness was readjusted to ensure that it was set to a moderate level in each individual, which is equivalent to a level of 60–70 dB SPL (e.g. Sandmann et al. 2009). This adjustment is important, since recent auditory brain imaging studies showed that the loudness can affect cortical activation (Zhou et al. 2022).

To have a measure of the subjective listening effort, participants were asked after every block of the experiment to rate the effort of understanding the syllables on a scale between zero (no effort at all) and five (very demanding). In addition, we asked the participants to rate the difficulty to perform the task on a similar scale (between zero (no effort at all) and five (very demanding)).

### Data Recording and Analysis

#### Behavioural Data: Auditory Oddball Task

The percentage of hit rates (hits) and individual mean response times (RT) of correct trials were analysed. Correct responses were defined as the occurrence of a button press in response to deviant syllables from 200 to 1200 ms following stimulus onset.

#### Electrophysiological Data: Recording and Data Processing

The EEG was continuously recorded with a BrainAmp DC amplifier from 30 active electrode sites, placed according to the extended 10/20 system (Brainproducts, http://www.brainproducts.com). An additional electrode was placed under the left eye for recording electrooculography (EOG), and the reference electrode was placed on the nose. The EEG was digitized at 1000 Hz, and the impedance was kept below 5 kΩ throughout the recording.

The data was analysed with EEGLAB (Delorme and Makeig, 2004) running in the MATLAB environment (R2020a; Mathworks). The EEG was downsampled to 500 Hz and offline filtered with a FIR-filter, using a high pass cut-off frequency of 0.1 Hz and a maximum possible transition bandwidth of 0.2 Hz (two times the cut-off frequency) plus a low-pass cut-off frequency of 40 Hz and a transition bandwidth of 2 Hz. For both filters, the Kaiser-window (beta = 5.653, max. stopband attenuation = -60 dB, max. passband deviation = 0.001) approach was used (Widmann et al. 2015). This approach maximises the energy concentration in the main lobe, thus averaging out noise in the spectrum and reducing information loss at the edges of the window (Widmann et al. 2015). Missing channels located over the region of the speech processor and transmitter coil were removed (mean and standard error: 0.6 ± 0.6 electrodes; range: 0–2 electrodes). The EEG data of the CI ear was segmented into epochs from − 100 to 400 ms relative to the stimulus onset, and it was baseline corrected (− 100 to 0 ms). Similar to previous studies, an independent component analysis (ICA) was then applied to the segmented data to identify the electrical CI artefact which spatially and temporally overlaps with auditory brain activity (Debener et al. 2007; Sandmann et al. 2010, 2009). After applying the ICA weights to the original (down-sampled and filtered (0.1–40 Hz) continuous EEG) data, all components that could be assigned to the electrical CI artefact were removed. Subsequently, the EEG datasets of both sides (CI ear: after first ICA-based artefact reduction; NH ear: original, i.e. down-sampled and filtered (0.1–40 Hz)) were merged and segmented into 2 s dummy segments. Segments exceeding an amplitude threshold criterion of four standard deviations were removed, and a second ICA was applied. All components assigned to ocular artefacts and other sources of non-cerebral activity were removed (Jung et al. 2000). Afterwards, the removed channels over the CI were interpolated using a spherical spline interpolation, a procedure still allowing good dipole source localisation of auditory event-related potentials (ERPs) in CI users (Debener et al. 2007; Sandmann et al. 2009). Only correct responses (hits for deviant syllables and correct rejections for standard syllables) were included for ERP analysis. Subsequently, a peak analysis of ERPs was performed on single-subject averages measured at different regions-of-interest (ROIs). We defined a frontocentral and a parietal ROI based on the grand average computed across all conditions and participants. The *frontocentral ROI* included the channels FCz, FC1, FC2, Fz and Cz and was used to analyse the N1 and P2 ERPs. The *parietal ROI* included the channels Pz, P3, P4, CP1 and CP2 and was used for the peak detection of the P3b component. For ERP quantification, individual peak amplitudes and latencies were measured by detecting the maximum and latency of ERP peaks in commonly used latency bands of the N1, P2 and P3b ERPs (Luck, 2014; Picton, 2010; N1: 80–160 ms; P2: 180–300 ms; P3b: 300–900 ms).

### Source Analysis

Cortical source activities were computed using the Brainstorm software (Tadel et al. 2011) and following the tutorial of Stropahl et al. 2018. Brainstorm applies the method of dynamic statistical parametric mapping of the data (dSPM; Dale et al. 2000). This method uses the minimum-norm inverse maps with constrained dipole orientations to estimate the locations of the scalp-recorded electrical activity of the neurons. It seems to localise deeper sources more accurately than standard minimum norm procedures, but the spatial resolution remains blurred (Lin et al. 2006). Prior to source estimation, the EEG data was re-referenced to the common average. Single-trial pre-stimulus baseline intervals (− 200 to 0 ms) were used to calculate individual noise covariance matrices and thereby estimate individual noise standard deviations at each location (Hansen et al. 2010). As a head model, the boundary element method (BEM) as implemented in OpenMEEG was used, providing three realistic layers and representative anatomical information (Gramfort et al. 2010; Stenroos et al. 2014). Source activities were evaluated in an a-priori defined auditory region-of-interest (ROI). The definition of the ROI was based on the Destrieux-Atlas implemented in Brainstorm (Destrieux et al. 2010). The used auditory ROI comprised four smaller regions of the original atlas (G_temp_sup-G_T_transv, G_temp_sup-Plan_tempo, Lat_Fis-post, S_temporal_transverse). These regions were combined using the “merge scouts” feature in Brainstorm and approximated Brodmann areas 41 and 42. Peak activation magnitudes and latencies within this ROI were extracted for each individual participant. The activation data is given as absolute values with arbitrary units based on the normalisation within the dSPM algorithm.

### Statistical Analyses

The subsequent statistical analysis was performed in R (Version 3.6.3, R Core Team 2020, Vienna, Austria). To address the four different research questions, the amplitudes and latencies of auditory ERPs were analysed separately on the sensor level (frontocentral ROI on head surface: N1, P2; parietal ROI on head surface: P3b) and on the source level (ERP source analysis: activation in ipsi- and contralateral auditory cortex at N1 latency). This was done by computing mixed ANOVAs with the between-subject factor “group” (proficient/non proficient CI users or left/right implanted patients) and the within-subject factors “stimulation side” (CI/NH), “stimulus type” (standard/deviant 1/deviant 2) and “hemisphere” (left/right). Significant interactions and main effects (p < 0.05) were followed-up by paired t-tests, and the Holm-Bonferroni approach was used for the correction of multiple comparisons (Holm, 1979). In the case of violation of spericity, a Greenhouse–Geisser correction was applied.

## Results

### Question 1: Can a Two-Deviant Oddball Paradigm Be Used to Objectively Evaluate the Speech Discrimination Ability in SSD CI Users?

#### Behavioural Results

The mean RTs of the nineteen SSD CI users were analysed with a two-way ANOVA, including the within-subject factors “stimulated ear” (CI, NH) and “stimulus type” (deviant 1, deviant 2). A significant main effect of “stimulated ear” (F_1,13_ = 23.82, p_adj_ ≤ 0.001, η^2^ = 0.22) was followed up by pairwise comparisons, revealing faster RTs when syllables were presented via the NH ear compared to the CI ear (p ≤ 0.001). A significant main effect of “stimulus type” (F_1,13_ = 8.56, p_adj_ = 0.012, η^2^ = 0.08) was followed up by pairwise comparisons, revealing faster response times for deviant 1 compared to deviant 2 (p = 0.04).

In contrast to the RTs, the hit rates showed a more complex pattern of results. While all participants could reliably discriminate deviant 1 from the standard sound (CI: 90.86 ± 0.07%, NH: 92.17 ± 0.05%), only some of the participants were able to reliably differentiate deviant 2 from the standard sound. Hence, the group of participants (including both the left- and right-ear implanted SSD CI users) was divided into two subgroups (proficient, non-proficient CI users) based on the median split in the behavioural performance (median of hit rate = 37.5%). In the following, these subgroups are referred to as proficient (performance > 37.5%) and non-proficient CI users (≤ 37.5%).

**Fig. 1:**
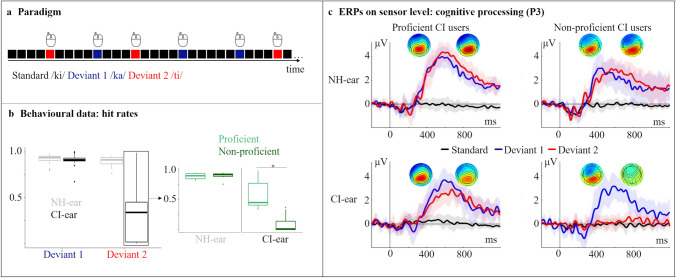
**a** Depiction of the oddball paradigm with frequent standard and two deviant syllables. **b** Hit rates for the NH- and the CI-ear separately for both deviant types plus the separation into two groups based on the hit rates for deviant 2. **c** ERPs showing the objectification of the groups by the P3 component with topographic plots. The shaded areas indicate the 95% confidence intervals. For illustration purposes, the ERPs are low-pass filtered (10 Hz). The left topography belongs to deviant 1 (blue line) and the right topography belongs to deviant 2 (red line), respectively. Time range for topographic plots: 555–575 ms

The hit rates of the participants were analysed using a three-way mixed ANOVA, with the between-subject factor “group” (proficient, non-proficient) and the within-subject factors “stimulated ear” (CI, NH) and “stimulus type” (deviant 1, deviant 2). A significant three-way interaction (F_1,17_ = 147.84, p_adj_ ≤ 0.001, η^2^ = 0.62) was followed up by simple two-way interactions and pairwise comparisons. As expected, there was a significant difference between the two groups for the *stimulation of the CI ear* (Fig. 1b): The proficient users had higher hit rates for deviant 2 when compared to the non-proficient users (p ≤ 0.001). By contrast, there was no group difference for the *stimulation of the NH ear*.

Comparing the response times between the proficient and non-proficient CI users revealed no significant difference between the two groups, neither for the CI ear nor for the NH ear.

#### ERPs: Proficient vs. Non-Proficient CI Users

The grand average ERPs from both the NH ear and the CI ear revealed an N1 and P2 response (Fig. 1c, Supplementary Fig. 1). In addition, the grand average ERPs showed a P3b ERP around 400–600 ms, which was observed in response to both deviant types in the proficient CI users, but which was restricted to deviant 1 in the non-proficient CI users (Fig. 1c).

In a first step of the ERP analysis, we focused on the N1 and P2 ERPs. We computed a two-way mixed ANOVA for the *N1 and P2 ERPs*, with the between-subject factor “group” (proficient, non-proficient) and the within-subject factor “stimulus type” (deviant 1, deviant 2). This was done separately for the stimulation over the CI ear and the NH ear. Regarding the *stimulation over the CI ear*, we found a main effect of “group” for the N1 amplitude (F_1,17_ = 4.16, p_adj_ = 0.057, η^2^ = 0.16) and the N1 latency (F_1,17_ = 5.68, p_adj_ = 0.029, η^2^ = 0.14), respectively. The pairwise comparisons revealed a significantly enhanced and prolonged N1 ERP for the non-proficient CI users when compared to the proficient CI users (averaged over all three stimulus types; amplitude: p = 0.02; latency: p = 0.03). No group differences were found in the P2 component (Supplementary Fig. 1a).

For the *stimulation over the NH ear*, we found no group effect for the N1 ERP, but a main effect of “stimulus type” (F_1,17_ = 42.99, p_adj_ = 0.001, η^2^ = 0.16), which was followed up by pairwise comparisons. This analysis revealed an enlarged N1 amplitude for deviant 2 compared to deviant 1 (averaged over both groups; p = 0.01). Regarding the P2 component, we found a significant two-way interaction between the factors “group” and “stimulus type” (F_1,17_ = 4.56, p_adj_ = 0.048, η^2^ = 0.03). The subsequent pairwise comparisons showed a greater P2 amplitude for deviant 2 for non-proficient CI users compared to the proficient CI users (p = 0.047).

In a second step, we focused on the P3b ERP. We computed a two-way mixed ANOVA with the between-subject factor “group” (proficient, non-proficient) and the within-subject factor “stimulus type” (deviant 1, deviant 2) separately for the CI ear and the NH. For *stimulation *via* the CI ear*, the analyses revealed a two-way interaction between the factors “group” and “stimulus type” (F_1,17_ = 6.13, p_adj_ = 0.002, η^2^ = 0.13), showing a significantly enhanced P3b amplitude for the proficient CI users specifically for deviant 2 when compared to the non-proficient CI users (p ≤ 0.001). No group difference was observed for the *stimulation *via* the NH ear* (p = 0.21). However, the stimulation via the NH ear showed a main effect for “stimulus type” (F_1,17_ = 8.6, p_adj_ = 0.009, η^2^ = 0.13), resulting in a significantly prolonged P3b latency for deviant 2 when compared to deviant 1 (p = 0.03). Furthermore, we found a significant positive correlation between the P3b amplitude and the hit rate of deviant 2 in both groups (proficient CI-users: R = 0.8, p = 0.009; non-proficient CI-users: R = 0.85, p = 0.002).

In sum, the results concerning question 1 revealed that ERPs, which are recorded in the context of a two-deviant oddball paradigm, show differences in initial sensory and later cognitive speech processing between different subgroups of SSD CI users. Specifically, non-proficient and proficient SSD CI users can be distinguished on the basis of the N1 and P3b amplitudes (for stimulation via the CI ear) as well as on the basis of the P2 amplitude (for stimulation via the NH ear). These findings suggest that the two-deviant oddball paradigm can be used to assess speech discrimination proficiency in SSD CI users.

### Question 2: Do SSD CI Users Show Differences in Speech Processing Between the CI Ear and the NH Ear?

Since not all participants were able to reliably identify deviant 2, this condition was removed for further statistical analyses.

#### Behavioural Results: CI Ear vs. NH Ear (SSD CI Users)

**Fig. 2:**
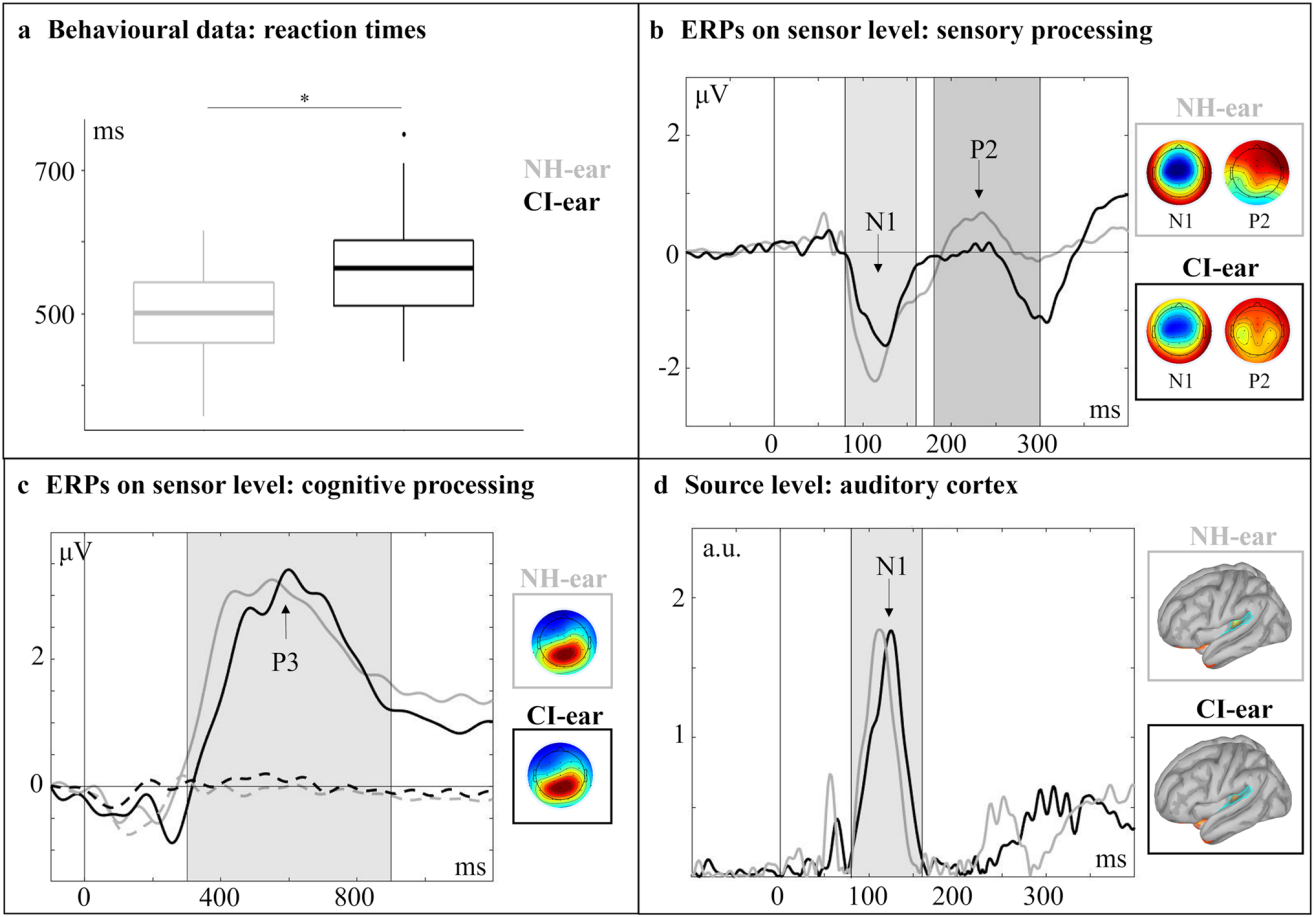
Comparisons between the NH- and the CI- ear in SSD patients on different levels. **a** Faster reaction times for stimulation over the NH-ear for deviant 1. **b** ERP-averages across standard and deviant 1 show a smaller and prolonged N1 component and a smaller P2 component for stimulation over the CI-ear. Time range for topographic plots: N1 = 100–130 ms (NH-ear)/110–140 ms (CI-ear), P2 = 220–250 ms. **c** ERPs to deviant 1 show a prolonged P3 component for stimulation over the CI-ear. Dotted lines represent responses to the standard syllable. Time range for topographic plots: 530–570 ms (NH-ear)/580–620 ms (CI-ear). **d** Source analysis shows a response in the auditory cortex at N1 latency which is delayed for stimulation via the CI. The blue area represents the used region of interest, red areas show activation. Time points for brain plots: 118 ms (NH-ear)/134 ms (CI-ear)

The comparison of the behavioural results for deviant 1 between the CI ear and the NH ear in SSD CI users (regardless of the side of implantation) showed no differences in hit rates but significantly faster response times (Fig. 2a) for the stimulation of the NH ear compared to the CI ear (t(18) = − 5.12, p ≤ 0.001, d = 0.92). Furthermore, the listening effort for syllable processing via the CI ear was significantly enhanced compared to the NH ear (t(18) = − 2.14, p = 0.047, d = 0.50).

#### ERPs on Sensor Level: CI Ear vs. NH Ear (SSD CI Users)

Figure 2 shows the ERPs in response to the standard and deviant 1, separately for the CI ear and the NH ear. The “stimulus type” (standard, deviant 1) x “stimulated ear” (CI, NH) ANOVA revealed a significant main effect of “stimulated ear” for the N1 amplitude (F_1,18_ = 16.54, p_adj_ ≤ 0.001, η^2^ = 0.13) and latency (F_1,18_ = 8.72, p_adj_ = 0.009, η^2^ = 0.13), respectively. The follow up pairwise comparisons revealed a smaller N1 amplitude (p = 0.001) and a prolonged N1 latency (p = 0.002) for the CI ear compared to the NH ear (averaged over both stimulus types). Similarly, the P2 amplitude revealed a significant main effect of “stimulated ear” (F_1,18_ = 12.10, p_adj_ = 0.003, η^2^ = 0.07), resulting in a significantly smaller amplitude for the CI ear than for the NH ear (p = 0.02). Paired t-tests between the P3b amplitudes and latencies of deviant 1 showed a prolonged latency for the CI ear compared to the NH ear (t(18) = − 27.27, p = 0.014, d = 0.62) but no ear difference in the P3b amplitude (Fig. 2c).

#### ERPs on Source Level: CI Ear vs. NH Ear (SSD CI Users)

Figure 2d shows the source activity separately for the two stimulation conditions (CI ear, NH ear) in the bilateral auditory cortex. Given that the ERP analysis on the sensor level did not reveal a significant effect of “stimulus type” (standard, deviant 1), the ERP analysis on the source level was restricted to the averages computed across the two stimulus types. The paired t-tests revealed a significantly delayed cortical response at N1 latency for the stimulation via the CI ear when compared to the NH ear (t(18) = 29.64, p = 0.008, d = 0.72). No significant difference was found for the amplitude of the source activity at N1 latency range.

#### Behavioural and ERP Results: Vocoded vs. Original Sounds (NH Listeners)

To evaluate whether the observed differences between the CI ear and the NH ear originate from the CI-related degradation of the stimuli (hypothesis 1) or from cortical plasticity (hypothesis 2), we compared the behavioural and ERP results between the two stimulus conditions “vocoded” and “original” syllables (separately for the two stimulus types: standard/deviant 1) within the group of NH listeners.

Regarding the *behavioural results*, the NH control group did not show any significant difference between the vocoded and the original syllables (Supplementary Fig. 2a). But the subjective listening effort for syllable processing with vocoded stimuli was significantly enhanced compared to the original stimulation (t(9) = − 2.42, p = 0.039, d = 0.78).

Concerning the *ERPs on the sensor level*, the supplementary Fig. 2b shows the waveforms of the NH control group separately for the “vocoded” and “original” stimulus conditions. The two-way ANOVA with the within-subject factors “condition” (vocoded/original) and “stimulus type” (standard/deviant 1) revealed no main effects and no significant interaction for the N1 ERP. However, the same ANOVA computed separately for the P2 ERP revealed a significant main effect of “condition” (F_1,9_ = 17.69, p_adj_ = 0.002, η^2^ = 0.15), resulting in a significantly larger P2 amplitude for the stimulation with vocoded syllables compared to the stimulation with the original syllables (p = 0.02). Finally, paired t-tests comparing the P3b ERP between the two stimulus conditions (vocoded/original) revealed no statistical difference in P3b amplitudes and latencies.

Concerning the *ERPs on the source level*, the supplementary Fig. 2d shows the source waveforms in the bilateral auditory cortex separately for the “vocoded” and “original” stimulus conditions. Paired t-tests comparing the two simulation conditions (vocoded/original) showed no statistical difference in the source activity at the N1 latency range, neither for the amplitude nor for the latency.

In sum, the findings on question 2 revealed that syllable processing via the CI ear—when compared to the NH ear —results in prolonged response times, enhanced subjective listening effort, and ERPs with reduced amplitudes (N1, P2) and prolonged latencies (N1, P2, P3b). These results suggest that the CI-related stimulus degradation leads to difficulties in speech processing in SSD CI users, not only at initial sensory but also at later cognitive processing stages.

### Question 3: Is there a Side-of-Implantation Effect on Speech Processing via the CI Ear in SSD CI Users?

#### Behavioural Results and ERPs on sensor level: Left CI vs. right CI (SSD CI users)

The group of nineteen SSD CI users was divided into two subgroups according to the implantation side. Ten participants were implanted on the left ear and nine on the right ear (Table 1). For the behavioural results (hit rates, response times) and the ERPs (amplitude and latency of N1, P2, P3b ERPs), we computed unpaired t-tests between the groups (left-implanted, right-implanted) separately for the CI ear and the NH ear. However, the results did not show any statistical differences between the left- and right-ear implanted SSD CI users.

#### ERPs on Source Level: Left CI vs. Right CI (SSD CI Users)

**Fig. 3:**
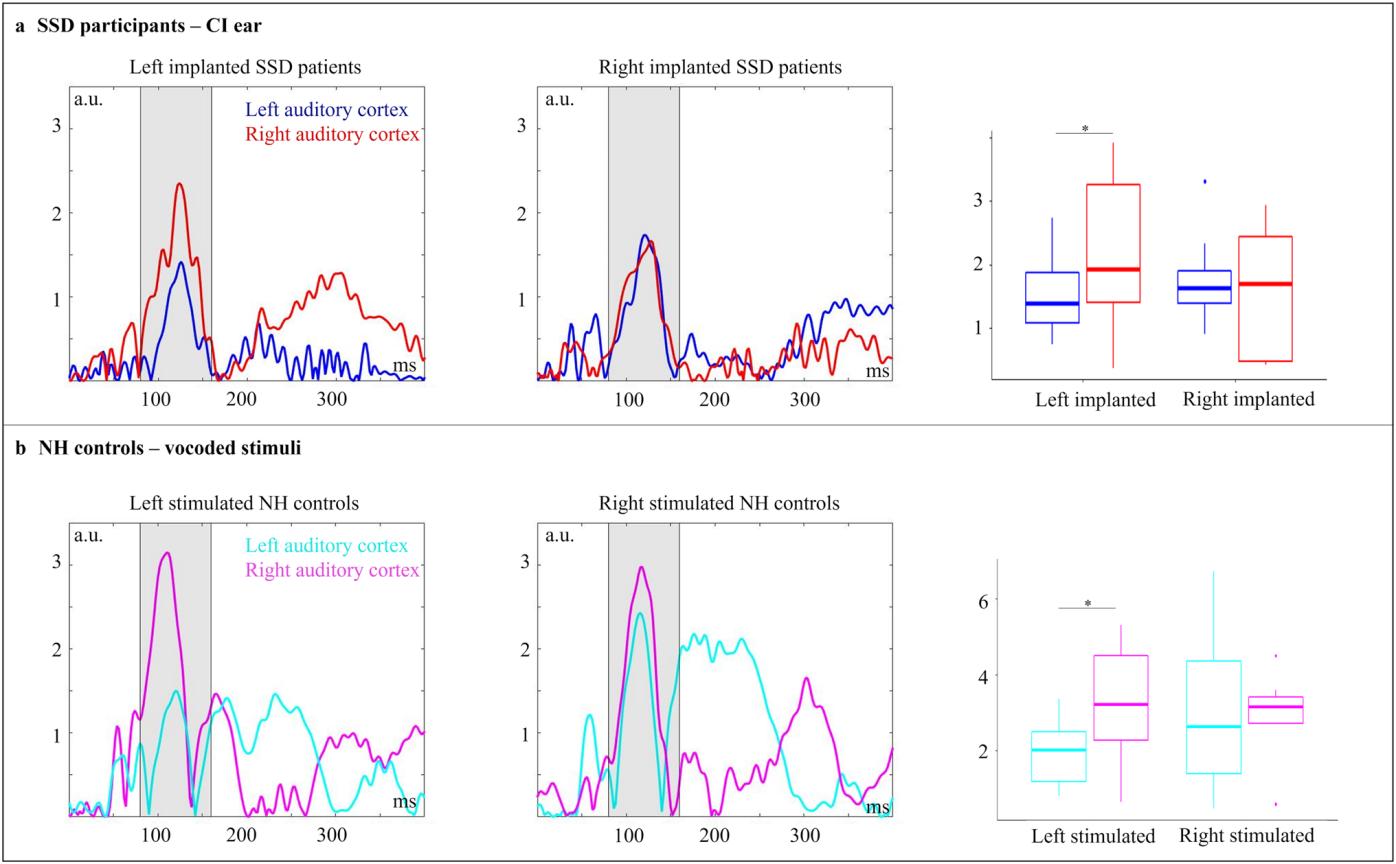
Activitiy in the left and right auditory cortex shows differences in the latency range of the N1 component for the groups. **a** Mean activities plus boxplots for all SSD patients, separated by implantation side, stimulated via the CI are shown. **b** Mean activities plus boxplots for all NH control subjects, separated by the stimulation side, for the stimulation with vocoded syllables are shown. Hemispheric asymmetries specifically for left implanted SSD patients and left stimulated NH controls, as indicated by enhanced N1 amplitude in the right compared to left auditory cortex

Figure 3a shows the activity in the left and right auditory cortex separately for left- and right-ear implanted SSD CI users when stimulated over the CI. A two-way mixed ANOVA with the between-subject factor “group” (left/right implanted) and the within-subject factor “hemisphere” (left, right) revealed a significant two-way interaction (F_1,17_ = 9.043, p_adj_ = 0.008, η^2^ = 0.17). Post-hoc t-test revealed a hemispheric difference for the left-implanted group (p = 0.031), with enhanced activity in the right than left auditory cortex. By contrast, the right-implanted group did not show a hemispheric difference in auditory-cortex activation.

#### Does the Side-of-Implantation Effect Arise from the Stimulus Degradation Through the CI or from Intra-Modal Plasticity in the Auditory Cortex?

To verify whether the observed hemispheric differences between the two SSD groups arise from the stimulus degradation through the CI (hypothesis 1) or from intra-modal plasticity in the auditory cortex (hypothesis 2), we compared the behavioural and ERP results between the two stimulus conditions “vocoded” and “original” syllables (separately for the two stimulation sides: left/right) within the group of NH listeners. We computed two-way ANOVAs including the within-subject factors “side of stimulation” (left/right) and “condition” (original/vocoded stimuli).

Regarding the behavioural results, we observed a significant main effect of “side of stimulation” for the hit rates (F_1,19_ = 14.26, p_adj_ = 0.001, η^2^ = 0.10), which was due to a higher hit rate for stimulation via the right ear when compared to the left ear (averaged over both conditions; p = 0.006). All other analyses revealed no statistical differences between the stimulation sides, neither in response times, nor in ERP measures (amplitudes and latencies of N1, P2, P3b ERPs).

Figure 3b illustrates the activity in the left and right auditory cortex for the NH control group when stimulated with vocoded syllables via the left and right ear. A two-way ANOVA with the within-subject factors “side of stimulation” (left/right) and “hemisphere” (left/right) revealed a significant two-way interaction (F_1,9_ = 9.85, p_adj_ = 0.012, η^2^ = 0.07). Post-hoc t-tests showed a hemispheric difference in auditory-cortex activation for the stimulation of the left ear (p = 0.028), with an enhanced activation in the right than left auditory cortex. By contrast, there was no hemispheric difference in auditory-cortex activation for the stimulation of the right ear.

In sum, the results concerning question 3 revealed no side-of-implantation effect on *behavioural* speech discrimination abilities in SSD CI users, although the NH listeners showed in general enhanced hit rates for the right-ear than the left-ear stimulation condition (i.e., regardless of whether the syllables were “original” or “vocoded”). In contrast to the behavioural findings, however, the ERP analyses revealed a side-of-implantation effect on auditory cortex functions for the SSD CI users, with enhanced hemispheric difference in auditory-cortex activation for the left-ear than the right-ear implanted individuals. A consistent pattern of hemispheric asymmetry was observed in the NH listeners, in particular when these individuals were tested with vocoded stimuli, that is, in approximated sound conditions. This suggests that the side-of-implantation effect on auditory-cortex asymmetry mainly originates from the CI-related degradation of the stimuli (i.e., confirmation of hypothesis 1).

### Question 4: Is there a Side-of-Stimulation Effect on Speech Processing via the NH Ear in SSD CI Users and in NH Listeners?

#### Behavioural Results and ERP Results on Sensor Level: Left Side vs. Right Side in SSD CI Users and NH Controls

We statistically compared the behavioural and ERP measures (for “original” syllables) for the NH ear of SSD CI users between the left and right implanted patients, hence the side-of-implantation effect on the NH ear. No differences were found for any behavioural measures, nor for the sensory ERP components (N1, P2). The t-test between the two groups for the higher-cognitive P3b component revealed a statistically significant difference in latency (t(16,98) = − 3.18, p = 0.005, d = 1.45), with a prolonged latency for the right SSD CI users (NH ear on the left side). The same analysis for the NH control group did not show any differences between the sides of stimulation.

#### ERPs on Source Level: Left Side vs. Right Side in SSD CI Users and NH Controls

**Fig. 4:**
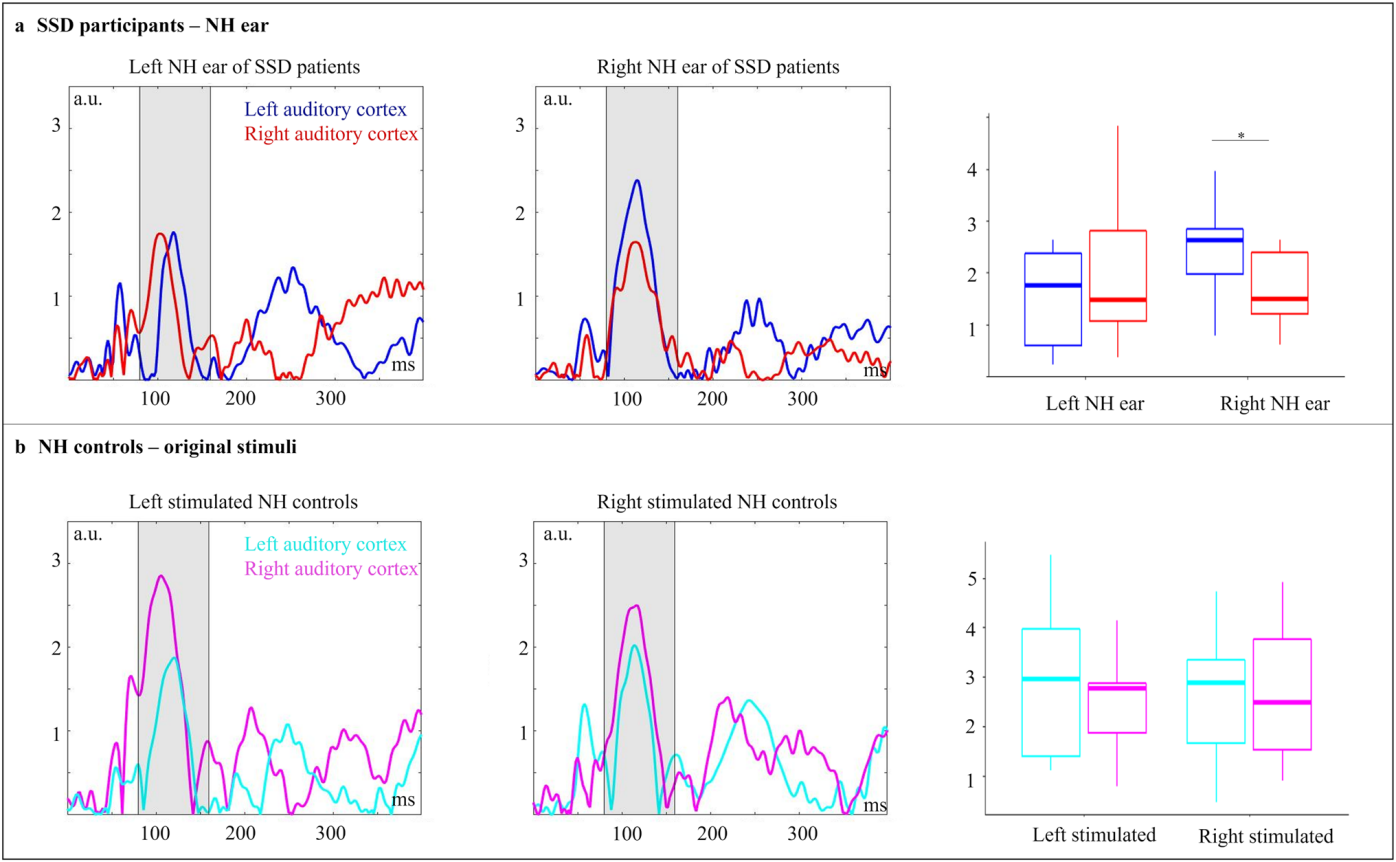
Activity in the left and right auditory cortex shows differences in the latency range of the N1 component for the groups. **a** Mean activities plus boxplots for all SSD patients, separated by implantation side, stimulated via the NH ear are shown. The left NH ear belongs to the right implanted SSD patients and the right NH ear belongs to the left implanted SSD patients. **b** Mean activities plus boxplot for all NH control subjects, separated by the stimulation side, for the stimulation with original syllables are shown. Hemispheric asymmetries specifically for right NH ear of the SSD patients, as indicated by enhanced N1 amplitude in the left compared to right auditory cortex

Figure 4a shows the activation of the left and right implanted SSD patients in the left and right auditory cortex when stimulated over their NH ear. A two-way mixed ANOVA with the between-subject factor “group” (left/right implanted) and the within-subject factor “hemisphere” (left/right) showed a significant two-way interaction (F_1,17_ = 5.91, p_adj_ = 0.026, η^2^ = 0.08). Post-hoc tests revealed a hemispheric difference in auditory-cortex activation for the stimulation via the right NH ear (left implanted group; p = 0.009), with greater activation in the left than right auditory cortex. By contrast, there was no hemispheric difference in the right-implanted SSD group when stimulated via the (left) NH ear. Additionally, a trend for a difference between the two SSD groups (when stimulated via the NH ear) was observed in the left auditory cortex, showing more activation for the stimulation of the right NH ear (left-implanted SSD users) compared to the left NH ear (right-implanted SSD users; p = 0.076).

The comparison of the two NH ears of the NH control group revealed no significant hemispheric differences in auditory-cortex activation, neither for the stimulation of the left nor the right ear (Fig. 4b).

In sum, the findings concerning question 4 revealed that stimulation via the NH ear in SSD CI users resulted in comparable *behavioural* speech discrimination abilities between left-ear and right-ear implanted individuals. However, the two groups of SSD CI users differed in the pattern of functional asymmetries in the auditory cortex. When stimulated via the NH ear, the left-ear implanted SSD CI users (NH ear on the right side) revealed a stronger auditory-cortex asymmetry than the right-ear implanted individuals (NH ear on the left side). The NH listeners in general showed a less pronounced side-of-stimulation effect. These results suggest a side-of-implantation effect on speech processing via the NH ear for SSD CI users, possibly caused by implantation-side specific alterations in the ipsilateral and contralateral auditory cortex as induced by temporary deafness and/or degraded sensory input after implantation (i.e., confirmation of hypothesis 2).

## Discussion

The present study compared event-related potentials (ERPs) between left-ear and right-ear implanted single-sided deaf (SSD) cochlear implant (CI) users on the one hand, and between these two patient groups and normal-hearing (NH) controls on the other hand. We used a two-deviant-oddball paradigm and ERP source analysis to evaluate differences in cortical speech processing between acoustic and electrical hearing as well as between proficient and non-proficient SSD CI users. Our results revealed that proficient and non-proficient CI users can be distinguished on the basis of N1 and P3b ERPs to speech sounds. Further, the results suggest that processing via the CI is more difficult than via the NH ear, as indicated by longer response times, higher subjective listening effort and ERPs with reduced amplitudes (N1, P2) and prolonged latencies (N1, P2, P3b; Finke et al. 2016, 2015; Henkin et al. 2009; Sandmann et al. 2009). Further, we found a stronger contralateral dominance of activation in the auditory cortex at N1 latency for left-ear than right-ear implanted SSD CI patients, regardless of whether these individuals were tested with the CI ear or the NH ear. A contralateral dominance for left-ear stimulation was also observed in the NH control group, which however was particularly present in the “vocoded” sound condition. We conclude that SSD CI users show a side-of-implantation effect on speech processing over both the CI and the NH ear. The next paragraphs focus on the four research questions addressed in the present study.

### Question 1: Can a Two-Deviant Oddball Paradigm Be Used to Objectively Evaluate the Speech Discrimination Ability in SSD CI Users?

Speech recognition ability after cochlear implantation is heterogeneous, meaning that many patients reach open-set speech recognition ability while others do not (Blamey et al. 2013). Such variability can also be observed in SSD CI users (Speck et al. 2021), as confirmed by the current results. Although all of our CI users were able to discriminate between the syllables /ki/ (standard) and /ka/ (deviant 1), several CI users showed difficulties in the discrimination of the syllable contrast /ki/ (standard) versus /ti/ (deviant 2). These findings were expected because the vowel contrast /i/ versus /a/ (deviant 1) is the highest possible contrast in the German language. By contrast, the consonant contrast /k/ versus /t/ (deviant 2) mostly refers to rapid spectral changes in the transition of the second formant (F2), making it more difficult to be distinguished. Not surprisingly, half of our CI users showed a hit rate below 37.5% for deviant 2, which confirms previous observations of impaired discrimination ability in CI users (Sandmann et al. 2010). It seems that difficulties with electrical hearing are caused by different factors, among them the limited spectral and temporal information provided by the implant, the spread of neural excitation in the cochlea, as well as physiological deficiencies in the auditory nerve (Drennan, 2008; Friesen et al. 2001; Kral, 2007; Nadol et al. 1989; Wilson and Dorman, 2008). Auditory deprivation not only reduces metabolism in the auditory cortex contralateral to the hearing-impaired ear (Speck et al. 2020) but also induces a reorganisation of the central auditory system, which may impair the cortical adaptation to the new artificial CI signal after implantation (Lee et al. 2001; Sandmann et al. 2012). Taken together, it seems that several individual factors contribute to the variability in speech discrimination ability observed in SSD CI users.

Our results on the stimulation via the CI ear also revealed a relationship between the (behavioural) auditory discrimination ability and objective ERP measures. CI users who were *better* able to discriminate between the syllables /ki/ and /ti/—here referred to as proficient CI users—showed a *reduced* and *delayed* N1 ERP when compared to the non-proficient CI users, who showed impaired syllable discrimination ability (Supplementary Fig. 1a: bottom left and right; Supplementary Fig. 1b). Importantly, the factor “age” cannot explain the reduced N1 ERPs in the proficient CI users, as the two groups had a comparable age, and a supplementary analysis revealed that the correlation between N1 ERPs and age was not significant (Supplementary Fig. 1c). Thus, our results suggest that the N1 ERP can distinguish between proficient and non-proficient CI users. Similarly, previous neuroimaging studies have suggested significantly different cortical activation patterns between lower and higher CI performers (Kessler et al. 2020), and an association between the recruitment of the auditory cortex and improvement in speech recognition ability over the first months after cochlear implantation (Sandmann et al. 2015).

Despite agreements with previous findings, the present ERP results indicate that the *non-proficient* CI performers revealed an *enhanced* N1 response when compared to the proficient CI performers, which however contradicts previous observations from positron-emission-tomography (PET) studies, reporting an *enhanced* cortical activation for individuals with *better* speech recognition ability (Giraud et al. 2001; Green et al. 2005). This discrepancy in results may be explained by differences in methodology, including the measurement technique (PET versus EEG), the task (passive versus active), the type of auditory stimuli (words versus syllables), and the tested groups of CI users (bilateral hearing loss versus SSD). In general, an enhanced N1 amplitude (observed in non-proficient CI users) indicates a larger population of activated neurons in the auditory cortex, a stronger synchronisation of this neural activity, or a combination thereof. Thus, the current results may point to an enhanced recruitment and/or more synchronised neural activity in the auditory cortex in the non-proficient as compared to the proficient CI users. It is likely that these functional alterations in non-proficient CI users reflect an enhanced allocation of attentional resources to process small acoustic changes in speech sounds in the context of a discrimination task.

It has been previously suggested for CI users with bilateral hearing loss that ERPs can be used to objectively evaluate the auditory discrimination ability (Henkin et al. 2009; Sandmann et al. 2010; Soshi et al. 2014). Most of these previous studies used an auditory oddball paradigm and focused on the P3b response, which is elicited by infrequent, task-relevant changes in stimuli, and which shows maximal amplitudes over parietal scalp locations (Polich and Comerchero, 2003). Different models exist to describe the P3b component (Verleger, 2020). For instance, the P3b has been proposed to be a correlate of decision making (O’Connell et al. 2012), and to reflect voluntary attention to the task-relevant target stimuli (Polich, 2007). However, the elicitation of such a response requires that the individual can distinguish the acoustical differences between the task-relevant and task-irrelevant events. Indeed, previous results with CI users have pointed to a connection between the behavioural discrimination ability and the P3b ERP. They have revealed that the P3b in response to deviant sounds is comparable between CI users (with bilateral hearing loss) and NH listeners if the acoustic cues are well distinguishable by the participants (Henkin et al. 2009). However, in situations with more difficult stimulus contrasts, the CI users’ P3b response was reduced in amplitude and prolonged in latency (Henkin et al. 2009). The current study with SSD CI users confirms these previous observations. Our proficient and non-proficient CI users were well able to discriminate the syllable contrast /ki/ versus /ka/ (deviant 1), both when tested via the NH ear and via the CI ear (Fig. 1b), and the P3b amplitude elicited to this syllable contrast was comparable across groups and stimulated ears (Fig. 1c). However, we found group differences in the P3b for the syllable contrast /ki/ versus /ti/ (deviant 2), specifically when the CI users were tested via the CI ear (Fig. 1c: bottom left and right). Proficient CI users, but not non-proficient CI users, showed a P3b in response to deviant 2. Additionally, we found significant correlations between the hit rates and the P3b amplitude in both groups, pointing to a direct connection between the discrimination ability and the P3b amplitude. Taken together, our results confirm previous studies by showing that the P3b is an appropriate ERP component to study higher-order cognitive processing in SSD CI users (Bönitz et al. 2018; Finke et al. 2016; Wedekind et al. 2021). Further, our results extend previous findings by demonstrating that the P3b can serve as an objective index for the behavioural speech discrimination ability in SSD CI users. Regarding the clinical application of the P3b, however, future studies should replicate and extend our results with more complex stimuli, for instance words. The use of similar stimuli in ERP recordings and common clinical test procedures (e.g., Freiburg monosyllabic word test) would allow even better comparability between the results of the electrophysiological P3b response and the behavioural word recognition ability obtained by speech audiometry.

Our results provide further evidence that ERP measures, in particular the N1 and the P3b, can differentiate between proficient and non-proficient CI users. This is consistent with other studies, reporting that ERPs measures—in particular the mismatch negativity (MMN; latency around 150–200 ms) and the P3b (latency around 300–650 ms) —can distinguish between CI users who have better versus lower abilities to discriminate speech sounds (Henkin et al. 2009; Turgeon et al. 2014). Thus, there is converging evidence that objective ERP measures can be used to assess behavioural speech recognition ability in CI users. However, the application of an EEG paradigm in the clinical context poses the challenge that the recording time should be as minimal as possible. Pakarinen et al. (2009) proposed a fast multi-feature paradigm for the recording of the mismatch negativity (MMN) to different speech sounds in the same recording session. Although this type of paradigm is very promising, the MMN is recorded in a passive condition and thus has a much smaller signal-to-noise ratio when compared to the P3b response. It is therefore reasonable to design a time-efficient *active* oddball paradigm, which allows to measure the more pronounced P3b in response to several speech stimuli and in a time short enough to avoid problems with vigilance, motivation, or restlessness of the patient. Our results are promising as they demonstrate that a two-deviant oddball paradigm is suitable to assess syllable discrimination proficiency in SSD CI users. Further, our findings extend previous reports by showing that cortical AEPs can be used in SSD CI users to objectify not only the detection (Távora-Vieira et al. 2018) but also the discrimination of speech sounds. Thus, our results suggest that this paradigm could be useful in the clinical context, as it allows the objective monitoring of the auditory rehabilitation in different acoustic dimensions after cochlear implantation. To extend our findings, which are limited to syllables, the paradigm should be extended to more complex speech stimuli such as words. Importantly, the objective ERP measures could indicate whether the custom setting of the CI is sufficient for detailed speech discrimination ability, and whether renewed adjustments in certain frequency ranges could be useful. This is particularly important for patients with an ambiguous constellation of behavioural results.

### Question 2: Do SSD CI Users Show Differences in Speech Processing Between the CI Ear and the NH Ear?

Our participants showed slower response times for the processing of syllables via the CI ear compared to the NH ear. This is consistent with recent work on SSD CI users, showing for the CI ear prolonged behavioural responses not only to sinusoidal tones (Bönitz et al. 2018) but also to words (Finke et al. 2016). Given the temporally and spectrally limited signal quality of the CI input, it is highly likely that these slower response times reflect enhanced difficulties to process the speech sounds via the CI ear when compared to the NH ear (Beynon et al. 2005; Groenen et al. 2001; Kelly et al. 2005). This interpretation is supported by our observation that SSD CI users report an enhanced listening effort for syllable processing via the CI ear as compared to the NH ear.

Similar to the behavioural results, we found an effect of stimulation type (acoustic versus electric) on ERPs, not only on the sensor level but also in the auditory cortex. For the CI ear, the ERPs (on the sensor level) were smaller in amplitude (N1, P2) and prolonged in latency (N1, P3b). These results are consistent with previous EEG studies on SSD CI users (Bönitz et al. 2018; Finke et al. 2016; Legris et al. 2018). CI-related effects on ERPs are also suggested by the current N1 source analysis, showing a prolonged cortical response to syllables when processed via the CI ear as compared to the NH ear (Fig. 2d). In sum, our ERP results are consistent with our behavioural observations since they suggest difficulties in speech processing via the CI ear, both at initial sensory and later cognitive processing stages. It is likely that the ERP differences between the CI ear and the NH ear are caused by CI-related stimulus degradation and/or cortical reorganisation as induced by temporary deafness and/or cochlear implantation (e.g., Sandmann et al. 2015).

To analyse the specific effect of CI-related stimulus degradation on speech processing, we compared the behavioural and ERP results of NH listeners between the two stimulus conditions “original” and “vocoded” speech sounds. The behavioural results did *not* reveal significant differences in hit rates and response times between the two sound conditions (Supplementary Fig. 2a). Further, N1 ERPs were comparable for “original” and “vocoded” syllables, both on the sensor level (Supplementary Fig. 2d) and in the auditory cortex (Supplementary Fig. 2d). These results indicate that the NH listeners could well distinguish the different syllable pairs, regardless of the CI-related stimulus degradation. Further, our results suggest that the cortical speech processing at N1 latency was not significantly affected by stimulus degradation in NH listeners. Given that noise-band vocoder simulations used in NH listeners allow a good approximation to the performance levels of CI users (Friesen et al. 2001), our results indicate that the attenuated and prolonged N1 ERP for the CI ear in our SSD patients cannot be explained by the degraded CI input alone. It seems more likely that this latency effect for the CI ear is at least partially caused by intra-modal plasticity in the auditory cortex of SSD CI users. Indeed, a previous prospective longitudinal study on CI users (with bilateral hearing loss) has shown that the N1 latency reduces over the first year of implant use and approaches the levels of NH listeners, but remains delayed, even after one year of CI experience (Sandmann et al. 2015). This observation indicates limitations in the capacity of the auditory cortex to adapt to the CI signal after implantation. Taken together, our results suggest that differences in speech processing between the CI ear and the NH ear in SSD CI users are at least partially related to limitations in cortical adaptation to the implant signal, causing difficulties in the sensory and cognitive processing when speech is perceived via the CI ear.

Unlike our findings about the N1 ERP, we observed an effect of stimulus degradation on the P2 response in the NH control group, with *enhanced* P2 amplitude for the “vocoded” sounds when compared to the “original” sounds (Supplementary Fig. 2b). With regards to the SSD group, however, speech processing via the CI ear resulted in a *smaller* P2 amplitude when compared to the NH ear. Two different mechanisms may account for this group specific differences in P2 amplitude modulation, in particular 1) training-related alterations of sound representation, and 2) allocation of attentional resources. Regarding the *first mechanism*, previous EEG studies have proposed that the training-related enhancement of the auditory P2 response represents an electrophysiological correlate of perceptual learning, memory, and training (Ross and Tremblay, 2009; Tremblay et al., 2001). It seems that P2 amplitude modulations are associated with cortical changes induced by repeated stimulus exposure rather than the learning outcome itself (Tremblay et al. 2014). Thus, our result of a smaller P2 response for the CI ear in SSD CI users can be explained by the fact that the NH ear—when compared to the CI ear—is more experienced and is more exposed to auditory stimuli as it is the dominant communication channel in these individuals. Regarding the *second mechanism*, previous studies with NH listeners have suggested that both the N1 and the P2 ERPs are susceptible to attention (Crowley and Colrain, 2004). An enhanced ERP amplitude at P2 latency can be explained by the attentional shift towards auditory stimuli (Picton and Hillyard, 1974), and seems to be associated with stimulus categorisation (García-Larrea et al. 1992). Following these previous studies, we interpret the larger P2 amplitude for “vocoded” stimuli in the NH control group as reflecting an enhanced allocation of attentional resources to process the degraded and unfamiliar stimuli. It seems that in this difficult listening condition, the NH listeners’ speech processing is not automatic but explicit and therefore needs the additional recruitment of cognitive resources to reconstruct the limited speech signal (Rönnberg et al. 2013; Zekveld et al. 2010). This interpretation is supported by the finding that the NH control group reported an enhanced subjective listening effort in the “vocoded” than the “original” sound condition.

### Question 3: Is there a Side-of-Implantation Effect on Speech Processing via the CI Ear in SSD CI Users?

It is currently not well understood, how the side of implantation affects the rehabilitation success in adult postlingually deafened SSD CI users. Recent results have pointed to a right-ear advantage for speech recognition ability in SSD CI users, independent from their pure tone thresholds (Wettstein and Probst, 2018). The authors have argued that this right-ear advantage in SSD CI users is mostly driven by the left-hemisphere dominance for speech processing. However, the current study could not replicate these previous findings, given that our behavioural results revealed no side-of-implantation effect on syllable processing. One may speculate that this lack of replication can be explained by the fact that the current study focused on syllables, whereas Wettstein and Probst (2018) presented four-syllabic numbers and monosyllabic words. The use of different stimulus types in the two studies obviously limits the comparability between the results. However, in addition to the EEG paradigm, our SSD CI users were also examined with standard clinical speech tests (Table 1). The results revealed no significant differences between left-ear and right-ear implanted SSD CI users regarding the pure-tone thresholds, the word recognition ability (assessed by the Freiburg monosyllabic word test) and the speech intelligibility (assessed by the Oldenburg sentence test). Thus, we speculate that the lack of a replication of a behavioural side-of-implantation effect may be related to the small sample size (used in the present study) in combination with the high variability in behavioural results observed in SSD CI users.

Our data revealed a significant hemispheric difference for the left-implanted participants, but not for the right-implanted participants, both for stimulation via the CI ear and the NH ear (Fig. 3a and Fig. 4a). For the stimulation via the *CI ear* (Fig. 3a), the left-implanted CI users showed a significantly enhanced activation in the right as compared to the left auditory cortex—referred to as contralateral dominance effect. For the stimulation via the *NH ear*, the left-implanted CI users (with a NH ear on the right side) showed a contralateral dominance effect as well, as indicated by a significantly enhanced activation in the left as compared to the right auditory cortex (Fig. 4a; see section “Question 4: Is there a side-of-stimulation effect on speech processing via the NH ear in SSD CI users and in NH listeners?” for a discussion of the side-of-implantation effect on the NH ear).

In contrast to children with SSD, who develop a normal lateralization to the contralateral auditory cortex when implanted at young age (Lee et al. 2020; Polonenko et al. 2017), not much is known about functional changes in the auditory cortex of postlingually deafened adult SSD CI patients. To discuss the observed differences in auditory-cortex asymmetry between our left- and right-ear implanted SSD CI users, it is important to keep two aspects in mind. First, the contralateral dominance of the auditory cortex has been described for monaural stimulation (Hine and Debener, 2007). In the human auditory system, the pathway from each ear to the contralateral cortical hemisphere consists of more nerve fibres than the pathway to the ipsilateral hemisphere (Rosenzweig, 1951). Therefore, monaural stimulation evokes stronger responses in the contralateral than in the ipsilateral hemisphere (Jäncke et al. 2002). Second, there is a relative hemispheric specialisation of the left and right auditory cortex for the processing of basic acoustic properties (Lazard et al. 2012a). Prior studies have suggested that the left auditory cortex is more proficient in the processing of fast temporal acoustic cues, which are largely contained in speech stimuli (Boemio et al. 2005; Poeppel, 2003). Conversely, the right auditory cortex seems to preferentially process slowly modulated signals and spectral aspects of sounds, which are largely contained in musical stimuli (Liegeois-Chauvel, 1999; Poeppel, 2003; Zatorre et al. 2002). Thus, the hemispheric differences during speech and music processing can be attributed to the *relative* specialisation of the two hemispheres for basic acoustic stimulus properties, in particular fast temporal versus slow spectrotemporal acoustic cues. It has been assumed that the auditory cortex’ preference for basic stimulus properties drives higher-order organisation for speech and music perception (Lazard et al. 2012a).

Our observation of a contralateral dominance effect for the left-ear implanted SSD CI users during speech processing seems to contradict previous results of left-hemisphere dominance for speech processing in NH listeners. However, a strong activation in the right auditory cortex in these patients can be explained by the combination of two factors. First, the left ear shows stronger projections to the contralateral than ipsilateral auditory cortex. Therefore, monaural stimulation of the left ear resulted in an enhanced activation in the right than left auditory cortex. Second, the CI processing remarkably reduces the quality of the speech sounds and affects the spectrotemporal properties of the presented syllables. Given the relative specialisation of the two hemispheres for basic acoustic stimulus properties, the CI-related stimulus degradation may have resulted in a relatively stronger right-than left-auditory cortex activation. Indeed, some of our SSD CI patients reported that the speech stimuli were perceived as more noise-like and less speech-like when presented via the CI than the NH ear.

Our results showed a contralateral dominance effect specifically for the left-ear but not for the right-ear implanted SSD CI users. This contrasts with the results of Sandmann et al. (2009), who found a stronger contralateral dominance effect for right- than left-ear stimulated CI users. This discrepancy in results can be due to different reasons. First, in the current study we used syllables, while Sandmann et al. (2009) used dyadic tones, i.e., musical sounds. Speech and musical stimuli are characterised by different acoustic properties. Given the relative specialisation of the left and right auditory cortex for basic stimulus properties (Poeppel, 2003; Zatorre and Belin, 2001), the discrepancies between previous and current results with regards to the pattern of cortical asymmetry could be explained by distinct stimulus properties, resulting in a different recruitment of the left and right auditory cortex. Another reason for discrepant findings between previous and current results is that the current study examined SSD CI users, whereas Sandmann et al. (2009) tested CI users with bilateral hearing loss. In contrast to the current study, the hearing ability of the second ear was reduced, and it was *not* matched between the left- and right-ear stimulated CI users. Given that auditory deprivation reduces the metabolism in the contralateral auditory cortex (Speck et al. 2020) and can induce cortical reorganization in the auditory cortex (Stropahl et al. 2017), the different pattern of hemispheric asymmetry in the auditory cortex might have arisen due to the confounding effect of the hearing loss in the second ear.

It may be argued that handedness is a factor confounding our results regarding functional hemispheric asymmetry, since the probability of a reversed lateralisation for language processing seems to be enhanced in left handers when compared to right handers (Hund-Georgiadis et al. 2002). However, previous studies using different methods have observed that the majority of right- *and* left-handed individuals show left-sided cerebral dominance for language processing, and only less than 10% of the left-handers show right-sided cerebral dominance for language processing (Khedr et al. 2002; Szaflarski et al. 2002). In addition, a supplementary analysis of our behavioural and ERP data revealed that the two left-handed CI-users lay within the normal range (as defined by mean ± 2 standard deviations) and therefore we conclude that the activity in the left and right auditory cortex was not confounded by the factor handedness.

Our results revealed a similar pattern of auditory-cortex asymmetry between the SSD CI users and the NH control group, when the latter group was presented with “vocoded” sounds (Fig. 3). Specifically, SSD CI users and NH listeners showed a contralateral dominance effect for the left-ear stimulation, with enhanced activation in the right as compared to the left auditory cortex. By contrast, no hemispheric difference was found for SSD CI users and NH listeners when they were stimulated via the right ear. Given these similarities between CI users and NH listeners (the latter tested with “vocoded” stimuli) and the fact that noise-band vocoder simulations allow a good approximation to sound conditions in CI users (Friesen et al. 2001), we conclude that the different pattern of contralateral dominance between left- and right-ear implanted CI SSD users is mainly driven by the CI-related stimulus degradation. Nevertheless, it is important to note that our sample size is limited, and the current study only allows conclusions to be drawn regarding the processing of syllables. Therefore, future studies should examine the functional asymmetry in the auditory cortex in further SSD CI users and for different auditory stimuli, in particular speech and musical sounds.

### Question 4: Is there a Side-of-Stimulation Effect on Speech Processing via the NH Ear in SSD CI Users and in NH Listeners?

A recent multicentre study has reported a significant difference in the hearing threshold between the NH ear of SSD patients and the NH ears of age-matched NH listeners (Arndt et al. 2019). This observation points to a poorer peripheral hearing capacity for the intact ear of SSD patients when compared to NH listeners. Importantly, these previous results suggest that SSD CI users show behavioural alterations not only in the CI ear but also in the NH ear. It seems that these alterations in the NH ear are *not* induced by cochlear implantation, given that the hearing threshold of the NH ear appears to be comparable at the times before and after implantation (Speck et al. 2021). Further, alterations in the NH ear of SSD CI users are not limited to the hearing threshold but can also show up in other auditory tests. For instance, Maslin et al. (2015) have reported that in SSD CI users the intact ear is better able to discriminate intensity differences, suggesting perceptual improvements as induced by cortical plasticity following unilateral deafness.

The present study did also reveal a significant difference between the NH ear of SSD CI users and the matched NH ear of NH listeners with regards to the hearing threshold, but not with regards to the behavioural performance and hit rates in the auditory oddball paradigm. Thus, our results can confirm alterations in the intact ear of SSD CI users, at least on the peripheral hearing capacity. But we could not confirm these alterations on the behavioural level in the oddball paradigm. Several reasons may account for the lack of comparability between the two results in the present study. In addition to the small sample size and the high variability in behavioural measures across participants, there are methodological discrepancies between the two measurements (Arndt et al. 2019; Maslin et al. 2015; Speck et al. 2021), in particular in terms of the task (pure-tone audiometry/intensity difference limens vs. auditory oddball task) and the stimulus material (pure tones vs. syllables). It may be speculated, that alterations in the NH ear of SSD CI users are stimulus- and task-selective and may be revealed only under specific conditions.

As far as we are aware, the present study is the first to compare the NH ears of SSD CI users and NH listeners in the context of an auditory oddball paradigm. The ERP source analysis revealed that *stimulation of the right NH ear* of (left-implanted) CI users induced an enhanced activation in the left as compared to the right auditory cortex —referred to as contralateral dominance effect (Fig. 4a top right). By contrast, the NH listeners—when stimulated on the right ear—showed no hemispheric difference in auditory-cortex activation, although on the descriptive level, the activation in the left hemisphere was enhanced (Fig. 4a bottom right). Regarding the *stimulation of the left NH ear*, both the (right-implanted) SSD CI users the NH listeners showed no activation differences between the right and left auditory cortex (Fig. 4a top left and bottom left). These results suggest that specifically the group of left-ear implanted SSD CI users shows cortical alterations for speech-sound processing when stimulated via the (right) NH ear (Fig. 4a top right). Interestingly, the same group also revealed an enhanced contralateral dominance effect when stimulated via the CI ear (Fig. 3a top left; see also section “Question 3: Is there a side-of-implantation effect on speech processing via the CI ear in SSD CI users?” for a discussion of this effect).

The enhanced hemispheric asymmetry for the stimulation of the *right NH ear* in (left-implanted) SSD CI users may be explained by alterations in the left auditory cortex for the processing of rapidly changing stimulus properties contained in speech stimuli (Boemio et al. 2005; Poeppel, 2003). It can be speculated that these improvements are induced by temporary unilateral deafness and/or electrical afferentation with a CI. These improvements may reflect an optimised left-hemispheric speech processing, which is particularly important in difficult listening conditions with reduced or degraded auditory input (processing via the CI ear), but which also affects the processing of the normal acoustic input (processing via the NH ear). Alternatively, but not mutually exclusive, previous animal studies have shown that unilateral deafness results in an enhanced ipsilateral activation, which is due to an increased number and/or enhanced excitability of neurons that are responsive to the intact ear (McAlpine et al. 1997; Mossop et al. 2000). Regarding the current results, the *reduced* hemispheric asymmetry for the stimulation of the *left NH ear* in (right-implanted) SSD CI users may be explained by the fact that SSD patients show enhanced afferent input from the (left) intact ear to the (left) ipsilateral auditory cortex (Maslin et al. 2015). Thus, the speech processing via the *left NH ear* in right-implanted SSD CI users might evoke a strong activation in the *left* auditory cortex, which counteracts the contralateral dominance effect for left-ear stimulation. Taken together, our results provide first evidence of a side-of-implantation effect on functional auditory-cortex asymmetry in adult postlingually deafened SSD CI users, which is not limited to the CI ear, but which is also shown for the NH ear. However, in order to gain a better understanding of the cortical changes in the intact ear of SSD CI users, future studies are required to examine whether a similar pattern of results on hemispheric asymmetries can be observed with other types of stimuli, for instance musical sounds and more complex speech stimuli.

## Limitations

One limitation of this study is the relatively small group size, which results in a low statistical power and therefore makes it difficult to draw conclusions that are transferable to the generality. However, we believe that our results point out important issues in single-sided deaf CI-users that are worth further research to support our findings of asymmetry in the auditory cortex depending on the side of implantation. Furthermore, we did not find correlations between our results of the EEG paradigm and the clinical speech tests reported in this manuscript. One important reason for this lack seems to be the fact that the syllable-discrimination task in the EEG paradigm (discrimination of phonetic contrasts) and the clinical speech tests (monosyllabic word test, Oldenburg Sentence Test (OLSA)) examine speech competencies on different linguistic levels. In particular, the comparison between the standard stimulus (/ki/) and the deviant 2 stimulus (/ti/) only relies on the consonant-contrast, which is very hard to discriminate for some CI-user, particularly in situations without any given context. A second reason could be our exploratory median split procedure. We used this procedure to divide our sample in equal group sizes, but it was not possible to get a division in *clearly poor* and *clearly high* CI performers. Further research should use more diverse and complex stimuli to differentiate the groups (proficient vs. non-proficient) on a more solid basis.

## Conclusions

The present study used an auditory oddball task to examine cortical speech processing of the CI ear and the NH ear of SSD CI users. Given that non-proficient and proficient SSD CI users could be distinguished based on the N1 and P3b amplitude, we conclude that the time-efficient two-deviant oddball paradigm can be used to assess speech discrimination proficiency in SSD CI users. Further, our results suggest that the observed differences in cortical speech processing between the CI ear and the NH ear in SSD CI users are (at least partially) caused by limitations in the cortical adaptation to the implant signal, which leads to difficulties in the sensory and cognitive speech processing for the CI ear. Finally, we found a side-of-implantation effect on auditory-cortex asymmetry for both the CI ear and the NH ear. We suppose that these side-of-implantation effects originate from CI-related degradation of the stimuli and cortical reorganisation as induced by temporary unilateral deafness and/or degraded sensory input after implantation.

## Supplementary Information

Below is the link to the electronic supplementary material.

Supplementary file1 (TIF 36206 kb)

Supplementary file2 (TIF 46049 kb)

## Data Availability

The datasets generated during and/or analysed during the current study are available from the corresponding author on reasonable request.
