# Supplementary figures and images for: Side-of-Implantation Effect on Functional Asymmetry in the Auditory Cortex of Single-Sided Deaf Cochlear-Implant Users
Source: Brain Topogr. 2022 Jun 7;35(4):431–52. doi: 10.1007/s10548-022-00902-3 (PMC9334411; doi:10.1007/s10548-022-00902-3)

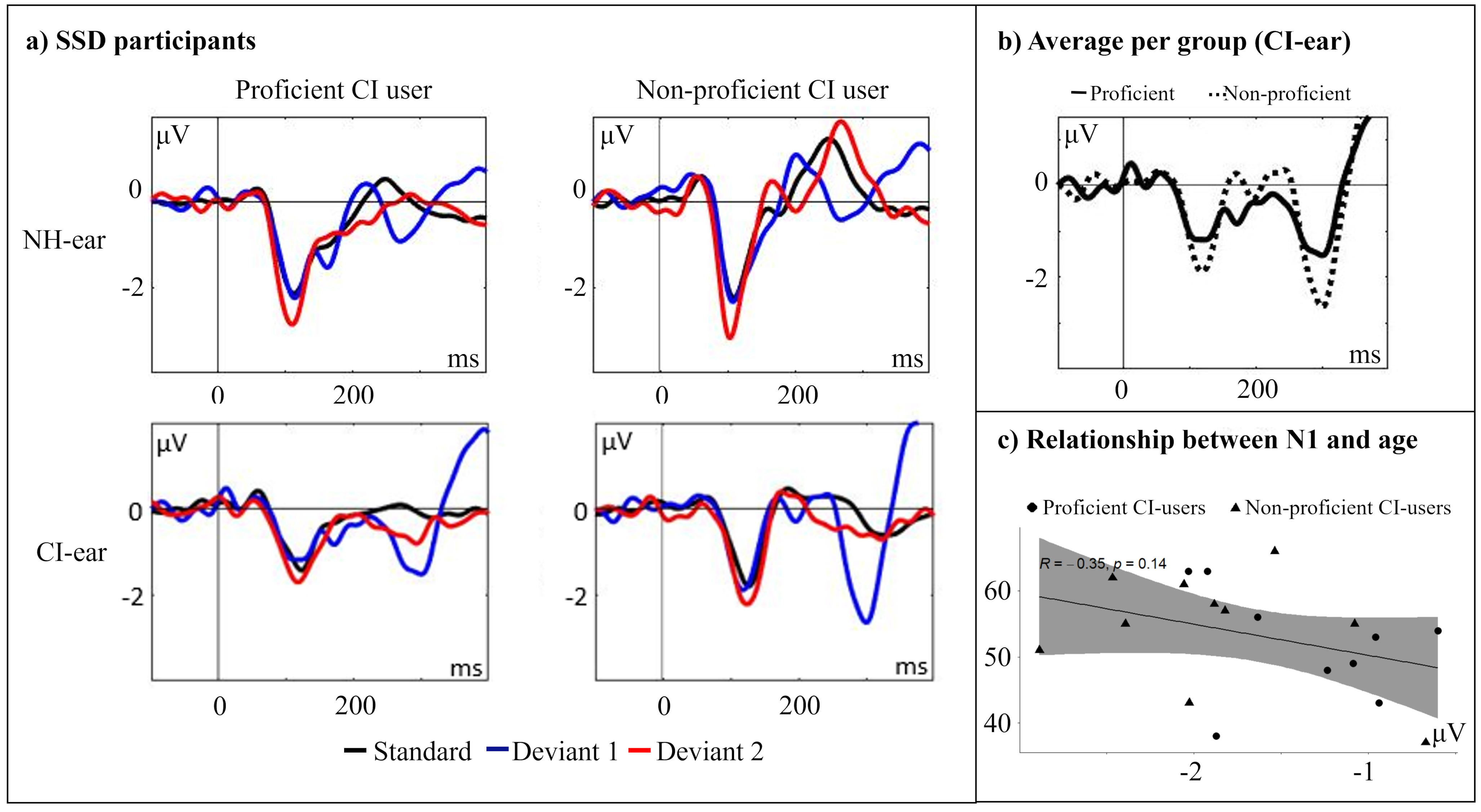

Supplement: Supplementary file 1 — Supplementary file1 (TIF 36206 kb) [file 10548_2022_902_MOESM1_ESM.tif]

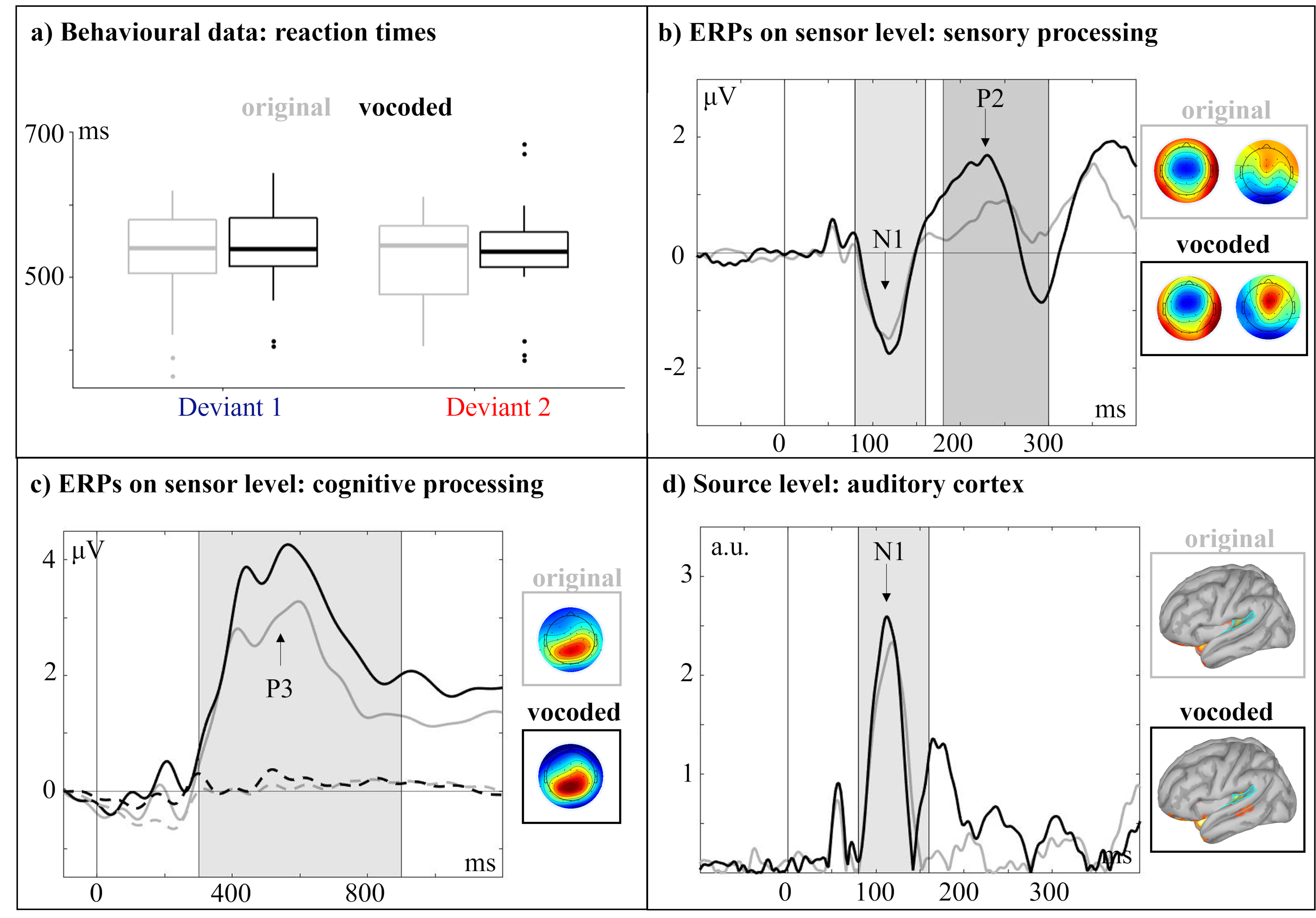

Supplement: Supplementary file 2 — Supplementary file2 (TIF 46049 kb) [file 10548_2022_902_MOESM2_ESM.tif]
